# Assessing Predictive Properties of Genome-Wide Selection in Soybeans

**DOI:** 10.1534/g3.116.032268

**Published:** 2016-06-17

**Authors:** Alencar Xavier, William M. Muir, Katy Martin Rainey

**Affiliations:** * Department of Agronomy, Purdue University, West Lafayette, Indiana 47907; † Department of Animal Science, Purdue University, West Lafayette, Indiana 47907

**Keywords:** bayesian methods, genomic selection, machine learning, SoyNAM

## Abstract

Many economically important traits in plant breeding have low heritability or are difficult to measure. For these traits, genomic selection has attractive features and may boost genetic gains. Our goal was to evaluate alternative scenarios to implement genomic selection for yield components in soybean (*Glycine max* L. merr). We used a nested association panel with cross validation to evaluate the impacts of training population size, genotyping density, and prediction model on the accuracy of genomic prediction. Our results indicate that training population size was the factor most relevant to improvement in genome-wide prediction, with greatest improvement observed in training sets up to 2000 individuals. We discuss assumptions that influence the choice of the prediction model. Although alternative models had minor impacts on prediction accuracy, the most robust prediction model was the combination of reproducing kernel Hilbert space regression and BayesB. Higher genotyping density marginally improved accuracy. Our study finds that breeding programs seeking efficient genomic selection in soybeans would best allocate resources by investing in a representative training set.

Soybean is a major crop used for human and animal consumption due to its ability to fix nitrogen and its unique seed composition (Board and Kahlon 2011; Chan *et al.* 2012). Genetic improvements in plant yield and quality can partially address increasing global demands for food quantity and quality. Unfortunately, genetic gains in soybean are often limited by its complex genomic properties (Hyten *et al.* 2007), resulting in low trait heritabilities. For such traits, genomic selection may outperform conventional breeding methods (Muir 2007) as well as having other promising and attractive features (Heffner *et al.* 2009; Jannink *et al.* 2010; Nakaya and Isobe 2012). Yet realistically, when resources are limited, many factors must be taken into account to optimize genetic gains (Meuwissen *et al.* 2001; Henryon *et al.* 2014; Poland 2015). Among the most important of these factors are: 1) training population size, 2) density of markers, and 3) prediction model. However, for several reasons, the genetic architecture of the population and the traits under consideration also affect these factors: linkage disequilibrium (LD) is population dependent (Hyten *et al.* 2007), traits differ in terms of heritability, and models differ in their assumptions and may not be effectively realized for some traits. As such, it is possible to determine these factors only by evaluating collected data pertaining to the populations and traits of interest.

Few studies have investigated genomic prediction in soybean (Jarquín *et al.* 2014; Xavier *et al.* 2016), and very little is known about the impacts on accuracy that these three factors have in this crop. We evaluated these factors using data collected from soybeans that were part of a nested association mapping (NAM) population. NAM is a next-generation experimental population, which is the result of crosses among single or multiple parent inbred lines followed by formation of recombinant inbred lines (Morrell *et al.* 2012). Guo *et al.* (2012) performed the first study in a NAM for genome-wide prediction (GWP), but focused on within-family prediction. However, a NAM is a complex structured population (Hamblin *et al.* 2011, Jannink *et al.* 2010) that can also be used for across-family prediction (Endelman *et al.* 2014), which is ideal for our objectives to determine the importance of: 1) training population size, 2) density of markers, and 3) prediction model on the accuracy of GWP in soybean.

## Materials and Methods

### Genetic material

The data used in this research came from SoyNAM, a soybean nested-association panel. The SoyNAM population (soynam.org) contains 5555 recombinant inbred lines (RIL) with maturity ranging from late maturity group II to early IV, derived from 40 biparental populations that share IA3023 as a common parent. Among the 40 founder parents, 17 lines are U.S. elite public germplasm, 15 have diverse ancestry, and eight are plant introductions. The genomic relationship among the lines is presented in Supplemental Material, File S1.

Lines were genotyped in the F_5_ generation with a 5k single nucleotide polymorphism (SNP) chip. The chip was designed using SNPs discovered after complete sequencing of the DNA of all parental lines and, as such, is not biased by sampling issues associated with rare variants (Daetwyler *et al.* 2013; Heslot *et al.* 2013). The pairwise linkage disequilibrium between SNPs is shown in File S1. We removed nonsegregating SNPs and variants with a minor allele frequency (MAF) lower than 0.15 (Jarquín *et al.* 2014). We also removed redundant markers, such as markers in full disequilibrium (LD), so that the genotypic data would represent natural bins (Xu 2013b). We coded the remaining genotypes as 0, 1, and 2 (Strandén and Christensen 2011), and imputed missing SNPs using random forest implemented in the R package missForest (Stekhoven and Bühlmann 2012).

### Field experimental design

Phenotypic data were collected from the SoyNAM population in 2013 and 2014 in West Lafayette, IN. We divided each of the 40 biparental families, with approximately 140 RILs each, into four blocks of 35 RILs each (40 families × 4 blocks =160 subblocks). The 160 subblocks were randomly assigned into the field, and the 35 RILs within each subblock were also randomized. In both years, lines were planted during the third week of May in two-row plots (2.9 m × 0.76 m), at a density of approximately 36 plants/m^2^.

### Phenotypes

We evaluated six traits: grain yield, days to maturity, plant height, pod number, node number, and pods per node. Grain yield was measured in grams per plot adjusted to a standard moisture for soybeans seeds of 13%. We collected days to maturity three times a week, with back and forward scoring of plots that matured in the intervals. Using a barcode ruler, we measured plant height in three plants per plot. We also counted the number of reproductive nodes and pods in the main stem during the reproductive stages R7–R8 (Fehr *et al.* 1971), measuring three and six plants per plot for 2013 and 2014, respectively, with the count of pods per node (P/N) being the ratio of these data points.

### Factors evaluated

#### Training population size:

We sampled subsets of 250, 500, 1000, 2000, 3000, and 4000 RILs at random as a training set to predict a validation set of 500 RILs that were not included in the training set.

#### Density of markers:

We tested subsets of the genotypic data as proposed by Meuwissen *et al.* (2001), using the whole panel, half panel, and quarter panel, corresponding to the 4077, 2039, and 1020 SNP markers, respectively. We formed the whole panel using all SNPs, the half panel by systematically choosing every other SNP, and the quarter panel by systematically choosing every fourth SNP.

#### Prediction models:

We tested the prediction performance of four additive models (parametric), two kernel models (nonparametric), and each combination of additive and kernel model. Combining models is a strategy of ensemble learning that uses the kernel to account for background genetic effects and the additive term to capture markers with large effects (Kärkkäinen and Sillanpää 2012). This practice is frequently used to incorporate pedigree information into prediction models (Henderson 1986; Muir 2007, de los Campos *et al.* 2009, Heffner *et al.* 2009), but instead we used the molecular data to represent the relationship among genotypes.

The additive models we evaluated included BayesA, BayesB, BayesC, and Bayesian least absolute shrinkage and selection operator (BLASSO), and two kernel models, reproducing kernel Hilbert space (RKHS), and genomic best linear unbiased predictor (GBLUP). GBLUP was based on a single linear kernel (Xu 2013a) and RKHS was based on multiple Gaussian kernels (de los Campos *et al.* 2010). We fitted the models using the BGLR package (Pérez and de los Campos 2014). In-depth theoretical bases for the model building are described elsewhere (Sorensen and Gianola 2002; Kärkkäinen and Sillanpää 2012; Gianola 2013; de los Campos *et al.* 2013; Pérez and de los Campos 2014).

### Method of evaluation

Predictive ability (PA) is defined as the correlations between observed (y) and predicted ($\hat{\text{y}}$) values computed through cross-validation (in which observations are not used to create the predictions). PA was based on 20 cross-validations for each combination of factors under evaluation. We estimated accuracy as PA divided by the square-root of heritability (Lehermeier *et al.* 2013).

Estimation of heritability (${\text{h}}^{2}$) for each trait-year employed restricted maximum log-likelihood (REML) using the EMMA algorithm (Kang *et al.* 2008) implemented in the R package NAM (Xavier *et al.* 2015a) to solve a mixed model with an additive genomic covariance structure. Thus, avoiding the use of different whole-genome regression models to compute heritability (de los Campos *et al.* 2015). The mixed model was defined in probabilistic terms as y∼$\text{N}(1\text{μ},ZGZ^{’}{\text{σ}}_{\text{a}}^{2}+I{\text{σ}}_{\text{e}}^{2})$, where y is the vector of phenotypes of a given trait by year, μ is the overall mean, Z is the incidence matrix of genotypes, G is the genomic kinship matrix (VanRaden 2008), ${\text{σ}}_{\text{a}}^{2}$ is the additive genetic variance, and ${\text{σ}}_{\text{e}}^{2}$ is the residual variance. We computed heritabilities as ${\text{h}}^{2}=\frac{{\text{σ}}_{\text{a}}^{2}}{{\text{σ}}_{\text{a}}^{2}+{\text{σ}}_{\text{e}}^{2}}$.

We limited the scope of the study to the impact of the defined factors upon accuracy. However, other measures of prediction properties have been suggested by Hastie *et al.* (2005) and Daetwyler *et al.* (2013), who used mean squared prediction error and prediction bias to identify problems with model fit.

### Data availability

Phenotypes of yield, height, and maturity along with genotypes are available through the R package SoyNAM (Xavier *et al.* 2015b). Imputed genotypes and other phenotypes are available upon request.

## Results

**Figure 1:**
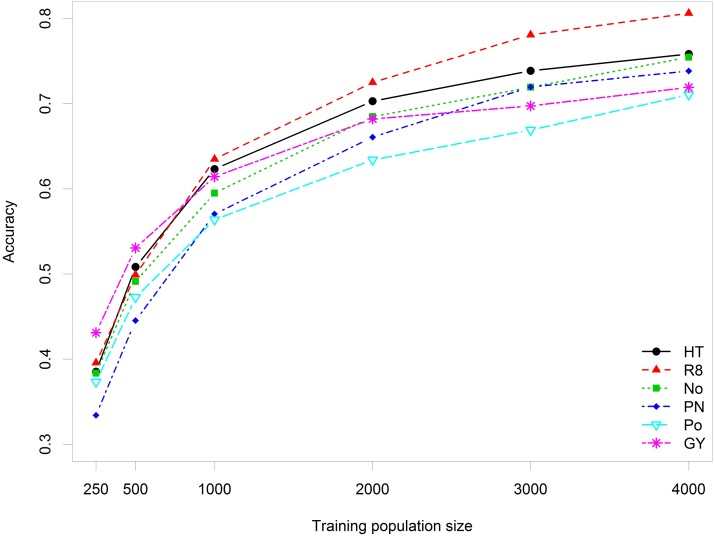
Effect of training population size on accuracy for six soybean traits. Plant height (HT), days to maturity (R8), number of reproductive nodes (No), pods per node (PN), number of pods (Po), and grain yield (GY).

Plant height was the most heritable trait, followed by grain yield and maturity, whereas the yield components appeared to be less heritable within the environment than yield itself. Figure 1 shows the effect of training population size on accuracy. Across traits, increasing the size of the training set from 250 to 4000 improved accuracy by 94.8% (from 0.384 to 0.747). Doubling the training population size increased accuracy by 9.1% on average. However, the improvements in accuracy decayed rapidly after 2000 individuals. Populations containing 1000–2000 individuals may represent the most cost-effective training sets, as gains become marginal for populations greater than 2000 individuals.

The number of markers was the least important factor for prediction in this study. Their effect on accuracy was marginal (1.33%). The use of all 4077 markers in comparison to 1020 increased accuracy from 0.600 to 0.607.

**Figure 2:**
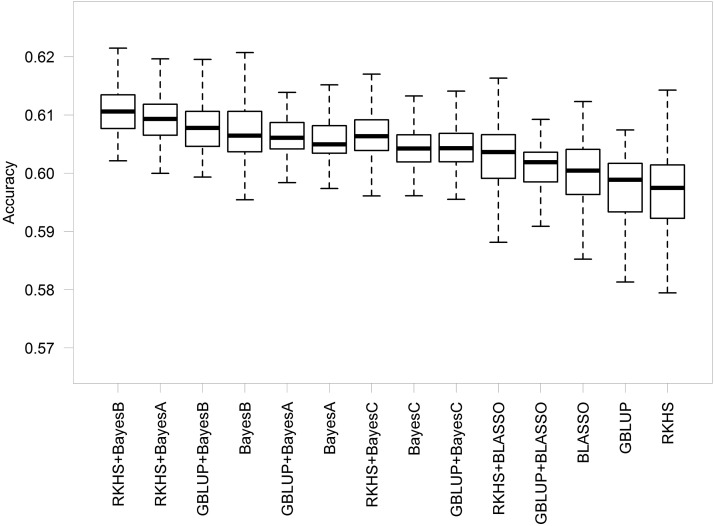
Boxplots of the accuracy of the genomic prediction models in soybeans tested in a variety of scenarios (*i.e.*, combinations of trait, number of SNPs, environment, and training population size). Whiskers represent the upper and lower limit, and the box represents the quartiles Q1 (25%), Q2 (median), and Q3 (75%). Models include additive methods (BayesA, BayesB, BayesC, and BLASSO), kernel methods (GBLUP and RKHS), and each combination of both. BLASSO, Bayesian least absolute shrinkage and selection operator; GBLUP, genomic best linear unbiased predictor; RKHS, reproducing kernel Hilbert space; SNP, single nucleotide polymorphism.

Figure 2 illustrates the performance of different prediction methods. Among the various prediction models, average accuracy ranges from 0.596 to 0.611. Further increases in accuracy were possible by selecting the optimum model for each scenario. The difference in accuracy among prediction models decreases as the training population size increases (File S1). The average improvement in accuracy achieved by selecting the optimal scenario model was 0.044 (3.84%). The best performing model for all traits was the combined model RKHS + BayesB (0.611).

We performed additional hypothesis testing using a *t*-test to compare between models to determine how specific model assumptions affect accuracy. Although differences between models were modest, models that included both an additive and kernel terms were significantly better than additive models alone (*P* = 0.009), and additive models were significantly better than kernel models (*P* < 0.001). Additive models combined with RKHS were significantly better than additive models combined with GBLUP (*P* = 0.004). Models with variable selection were significantly better than their all-included counterparts (*P* < 0.001).

## Discussion

Genomic estimated breeding values (GEBVs) generated through whole-genome regression (WGR) enable breeding programs to speed up the breeding process (Heffner *et al.* 2009; Endelman *et al.* 2014). Selection based on GEBVs is more reliable than that based on phenotypes alone or the traditional Quantitative Trait Loci (QTL) pyramiding (Nakaya and Isobe 2012), and it provides more genetic gains over the long term when compared to pedigree-based breeding values (Muir 2007). GEBVs are used to select unphenotyped material (Heffner *et al.* 2009), to perform more accurate selection of advanced lines (Endelman *et al.* 2014), to incorporate useful germplasm (Chung *et al.* 2014), to monitor the loss of genetic diversity (Henryon *et al.* 2014), and to select parental combinations for crosses (Mohammadi *et al.* 2015). Yet studies of GWP are important because the methodology for GEBV estimation is not fully understood (de los Campos *et al.* 2015), and the best prediction model varies among traits and from crop to crop (de los Campos *et al.* 2013). In addition, the contribution of genotyping density, population size, and phenotyping to genomic prediction is not clear when applied to real data (Wimmer *et al.* 2013). Prediction studies often provide conflicting results that vary according to the genetic basis of the population under evaluation (de los Campos *et al.* 2013).

### Environmental factors

There are many strategies by which breeders can maximize genetic gains (Henryon *et al.* 2014). Robust breeding values rely on accurate phenotypic data collection and good environmental control by employing replications, checks, neighbor plot information, field plot techniques, and a well-planned field design (Heffner *et al.* 2009; Endelman *et al.* 2014). Similar results obtained in both the 2013 and 2014 environments (Table 1) indicate a stable level of genetic control across seasons. In this experiment, yield, height, and maturity were more heritable than yield components. We attribute the low heritability of yield components to their sensitivity to microenvironmental variation (Board and Kahlon 2011).

**Table 1:** Genomic heritability (h^2^), average predictive ability [Cor(y, ^y)], and accuracy in two environments (2013 and 2014) for six soybean traits

Trait	h^2^		$\text{Cor}(\text{y},\hat{\text{y}})\;$		Accuracy	
	2013	2014	2013	2014	2013	2014
HT	0.522	0.478	0.459	0.418	0.635	0.604
R8	0.374	0.317	0.398	0.355	0.650	0.630
No	0.307	0.259	0.334	0.309	0.603	0.607
PN	0.238	0.189	0.275	0.258	0.563	0.593
Po	0.264	0.253	0.283	0.296	0.552	0.589
GY	0.494	0.409	0.423	0.399	0.602	0.623
Mean	0.366	0.317	0.362	0.339	0.601	0.608

h2, genomic heritability; Cor(y,$\hat{\text{y}}$), average predictive ability; HT, plant height; R8, days to maturity; No, number of reproductive nodes; PN, pods per node; Po, number of pods; GY, grain yield.

Replicated trials are not commonly used in GWP and mapping studies (Jannink *et al.* 2010). In this study, all cross-validations were performed within environment, which can affect the heritability and predictive ability in different ways (Endelman *et al.* 2014). In addition, genome-based heritability estimates in structured populations often provide lower values than pedigree-based estimates (Dekkers 2012). Nevertheless, results indicate that even low-heritable traits still provide reasonable accuracy. Muir (2007) pointed out that traits with low heritability have greater potential to be exploited. On the other hand, if accuracy is interpreted as the amount of genetic gains that genomic selection can exploit, less heritable traits with genomic data may provide accuracy comparable to more heritable traits.

Soybean yield components are commonly used as covariates in production systems to predict grain yield. The same approach should not be applied in genomic selection models targeting the genetic improvement of grain yield, because genetically correlated traits share additive genetic background (Valente *et al.* 2015). For breeding purposes, yield-component information is more suitable for enhancing grain yield, using multivariate models that accommodate the genetic relationship among traits (Rosa *et al.* 2011). In addition, low-heritable traits are favored by multivariate schemes (Sorensen and Gianola 2002).

### Training population size

Training population size had the greatest impact on accuracy (Figure 1), which can determine the success of GWP. Two main properties of the training set are known to be critical to GWP: its relatedness to the validation set (Habier *et al.* 2007) and the population size (Nakaya and Isobe 2012). Good training sets must be representative of the germplasm under evaluation to capture the population structure and have a population size sufficient for an accurate estimation of allelic effects (Jannink *et al.* 2010). SoyNAM is a finite population with constrained structure, allowing the model calibration to become more accurate as the training set size increases. The remaining question is: what population size is sufficient for good prediction?

Quantitative traits are mostly controlled by alleles of small and medium effect, so that larger training sets will increase the signal-to-noise ratio (Muir 2007) and provide better learning properties (Okser *et al.* 2014). This potentially allows more accurate allelic effect estimates by minimizing the Beavis effect at the whole-genome level (Xu 2003). Besides the quantity of the training population, the quality also determines the success of prediction and long-term breeding (Bastiaansen *et al.* 2012). The quality of the training set with regard to its genetic variability depends on the effective population size, which is always smaller than the total number of genotypes. Soybean and other self-pollinated species often suffer from reduced effective population size because of their reproductive nature (Cowling *et al.* 2015; Hamblin *et al.* 2011) and narrow genetic basis restricted to elite germplasm (Hyten *et al.* 2006).

A sufficiently large training population size is also required when the ultimate goal is to perform selection of unphenotyped material (Heffner *et al.* 2009). When the training set is part of a breeding population being phenotyped and selected over generations, increasing the training population size is always beneficial to increase genetic gains (Bastiaansen *et al.* 2012; Hamblin *et al.* 2011; Muir 2007). In some cases, training population size is also critical for the choice of prediction model (Bastiaansen *et al.* 2012).

### Prediction model

Varying the parameterizations of genomic information in prediction models to suit the particular genetic architecture of a trait can enhance prediction accuracy (Bastiaansen *et al.* 2012; Dekkers 2012; de los Campos *et al.* 2013). Pérez-Rodríguez *et al.* (2012) compared the performance of additive and kernel methods on two wheat traits across several environments, showing that BayesB better predicted yield grain while RKHS was the best model to predict days to heading. Similarly, Zhong *et al.* (2009) reported that GBLUP and BayesB are each better suited to different barley traits. Our results indicate that fitting parametric and semiparametric terms together provides a more robust prediction of soybean traits than either additive or kernel methods alone.

For the traits under evaluation, the combination of BayesB and RKHS provided the highest accuracy. Kärkkäinen and Sillanpää (2012) reported a synergy between BayesB and the semiparametric term, perhaps because kernels account for the relationship among individuals (Okser *et al.* 2014), while BayesB captures QTL in disequilibrium with markers in an additive fashion. The combination of a RKHS with BayesB includes flexible assumptions that account for different genetic interactions. RKHS enables the model to capture some level of epistasis (González-Camacho *et al.* 2012; Howard *et al.* 2014) with no assumptions about additive inheritance (de los Campos *et al.* 2009; Gianola *et al.* 2009), and BayesB allows markers to have large and/or null effect (Habier *et al.* 2011).

The decision to include kernels (pedigree or genomic) to account for the polygenic term in the prediction model depends on many factors, such as the marker density (Heffner *et al.* 2009), availability and complexity of pedigree data, and genetic architecture of the trait (de los Campos *et al.* 2013). Our results indicate that there is no advantage in utilizing kernel methods in this soybean population, in contrast to reports from simulations and studies with wheat and maize (González-Camacho *et al.* 2012; Pérez-Rodríguez *et al.* 2012; Howard *et al.* 2014).

In combined models, RHKS is a better complementary method than GBLUP. RKHS accounts for different levels of relationships among individuals due to the nonlinear nature of Gaussian kernels (de los Campos *et al.* 2010; González-Camacho *et al.* 2012). In kernel methods, markers are informative regardless of whether they are linked to any QTL (Habier *et al.* 2007), whereas null effect markers would harm any additive model incapable of performing efficient variable selection.

Our results indicate that models with a variable selection term provide better predictions. Efficient prediction often relies on consistent variable selection (Okser *et al.* 2014), especially in soybeans and other species that have a small genome, large LD blocks, and restricted diversity (Hyten *et al.* 2006, 2007; Chung *et al.* 2014), which together cause markers to present severe multicollinearity. Wimmer *et al.* (2013) showed that variable selection improves prediction in the presence of major effect genes and large populations in rice, wheat, and *Arabidopsis thaliana*.

### Genotyping density

Higher genotyping density does not always increase accuracy (VanRaden *et al.* 2011), and subsets of the genotypic data sometimes outperform the entire dataset (Erbe *et al.* 2012). Xu (2013b) observed that artificial bins that compress genotypic information into fewer parameters could provide more accurate results than natural bins.

For the SoyNAM population, 1020 markers are enough to provide a consistent prediction, while higher density genotyping provides only marginal gains in PA. This result is likely associated with soybean’s genomic properties, such as the existence of large disequilibrium blocks (Hyten *et al.* 2007) and the uneven distribution of SNPs in the soybean genome (Li *et al.* 2014).

The importance of larger SNP panels increases when the population structure is unknown, the number of selection cycles increases, and the LD between the QTL and marker decays (Bastiaansen *et al.* 2012; Daetwyler *et al.* 2013). In terms of allocating resources, our results support increasing population size over higher genotyping density, and using replicated trials when the number of genotypes in the training set is already sufficiently large (Lorenz 2013).

### Conclusions

By comparing the accuracy associated with multiple factors, we showed that training population size is the main limiting factor for accuracy in soybeans. However, the rate of improvement decreased rapidly above 2000 individuals, suggesting that an optimal population size exists for the dataset in a study of between 1000 and 2000. The choice of prediction models was not unique for all scenarios. The best prediction model for this soybean population was the combination of RKHS and BayesB, which accommodates markers with large and null effect, also capturing some level of epistasis.

The value of next-generation populations to exploit new genomic frontiers is not limited to genome-wide associations. Prediction experiments based on real data provide an important insight for resource allocation, planning, and decision making in soybean breeding programs that aim to optimize genetic gains through genomic selection.

Soybean is a crop of worldwide importance that has shown limited rates of genetic gains. The use of genomic information through prediction models is a possible solution for more effective genetic improvement. In this study, we showed how genomic prediction acts in complex soybean traits in a variety of scenarios. The study shows how different factors affect the estimation of genomic values within environments. We believe that future directions for genome-wide prediction studies in soybeans should evaluate predictions across environments and across generations, as well as the optimal prediction procedures for genetic panels in ongoing selection.

## Supplementary Material

Supplemental Material
