# Supplementary material for: Assessing Predictive Properties of Genome-Wide Selection in Soybeans
Source: G3 (Bethesda). 2016 Jun 17;6(8):2611–6. doi: 10.1534/g3.116.032268 (PMC4978914; doi:10.1534/g3.116.032268)
Supplement: Supplemental Material [file supp_6_8_2611__index.html]

Assessing Predictive Properties of Genome-Wide Selection in Soybeans — Assessing Predictive Properties of Genome-Wide Selection in Soybeans — Supplemental Material 

# Assessing Predictive Properties of Genome-Wide Selection in Soybeans

## Supplemental Material for Xavier, Muir, and Rainey, 2016

**Files in this Data Supplement:**

- File S1 - Additional description of populations and genomic patterns. (.docx, 1.21 MB)
