# Supplementary material for: Assessing Predictive Properties of Genome-Wide Selection in Soybeans
Source: G3 (Bethesda). 2016 Jun 17;6(8):2611–6. doi: 10.1534/g3.116.032268 (PMC4978914; doi:10.1534/g3.116.032268)
Supplement: Supplemental Material [file supp_g3.116.032268_FileS1.docx]

**File S1**

**Additional description of populations and genomic patterns**


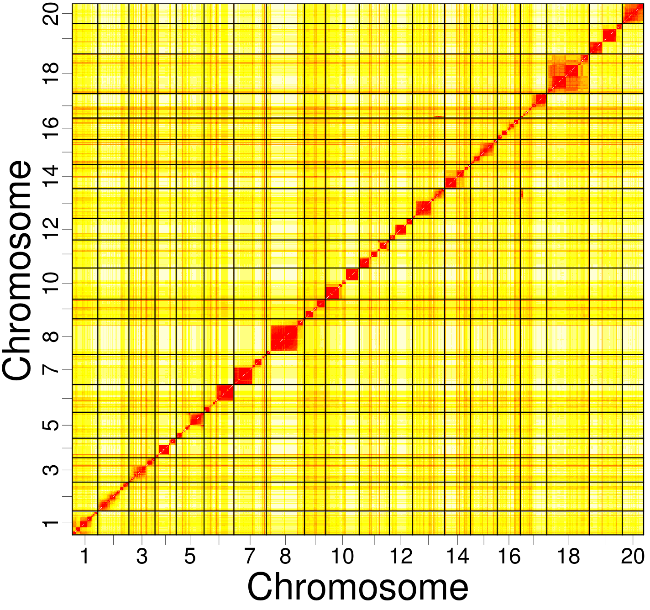


**Fig. 1** SNP panel pairwise linkage disequilibrium among the 4077 markers in terms of $r^{2}$ in the SoyNAM population.


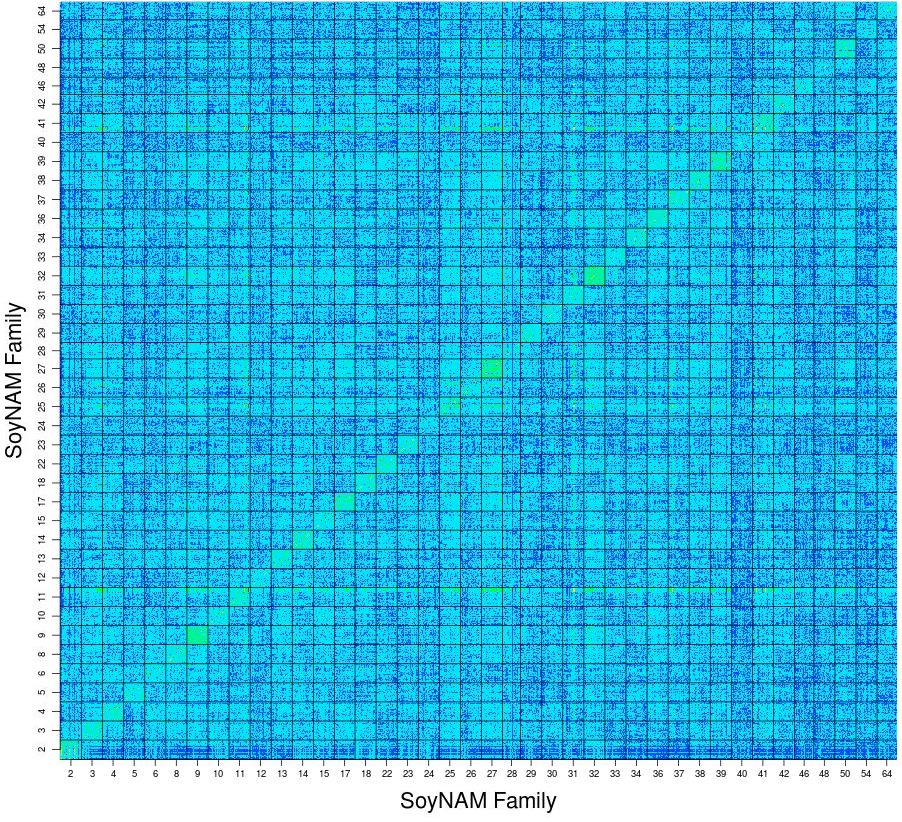


**Fig. 2** Heatmap of the genomic relationship matrix of the SoyNAM population with delimitations indicating family.


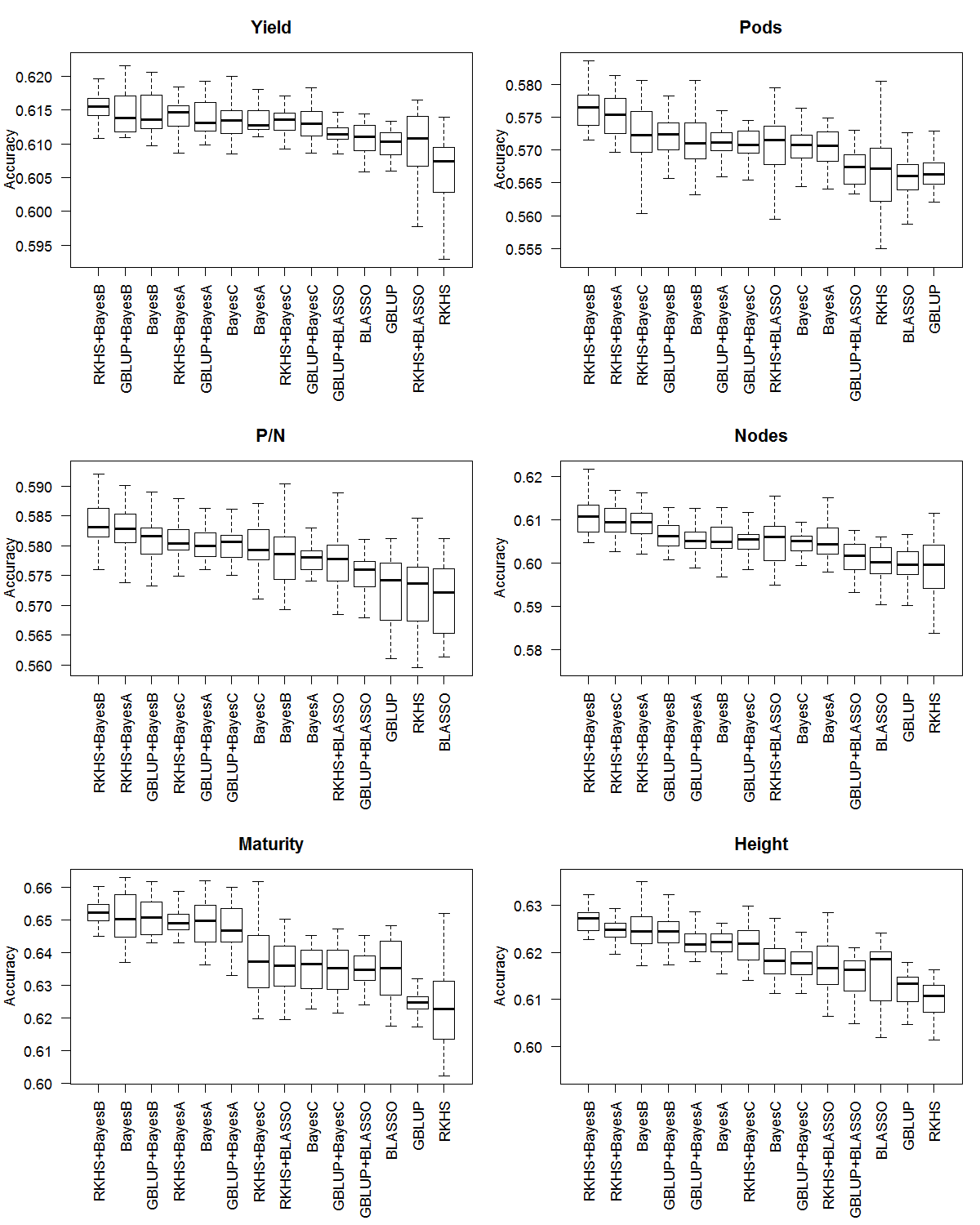


**Fig. 3** The boxplot of accuracy of different genomic prediction models in soybeans across different scenarios (ie. combinations of trait, number of SNPs, environment and training population size). Whiskers represent the upper and lower limit, and the box represents the quartiles Q1 (25%), Q2 (median) and Q3 (75%). Models include additive methods (BayesA, BayesB, BayesC, BLASSO), kernel methods (GBLUP, RKHS) and each combination of both.

**Table 1** Average accuracy of different prediction models for different soybean traits, comprising plant height (HT), days to maturity (R8), number of reproductive nodes (No), pods per node (PN), number of pods (Po), and grain yield (GY). Models include additive methods (BayesA, BayesB, BayesC, BLASSO), kernel methods (GBLUP, RKHS) and each combination of both.

|  | GY | Po | PN | No | R8 | HT |
| --- | --- | --- | --- | --- | --- | --- |
| BayesA | 0.613 | 0.570 | 0.578 | 0.605 | 0.649 | 0.622 |
| BayesB | 0.614 | 0.572 | 0.579 | 0.606 | 0.651 | 0.625 |
| BayesC | 0.614 | 0.570 | 0.580 | 0.605 | 0.635 | 0.618 |
| BLASSO | 0.611 | 0.566 | 0.571 | 0.599 | 0.635 | 0.615 |
| GBLUP | 0.610 | 0.566 | 0.572 | 0.599 | 0.625 | 0.612 |
| GBLUP+BayesA | 0.614 | 0.571 | 0.580 | 0.606 | 0.648 | 0.622 |
| GBLUP+BayesB | 0.615 | 0.572 | 0.582 | 0.607 | 0.651 | 0.624 |
| GBLUP+BayesC | 0.613 | 0.571 | 0.580 | 0.605 | 0.635 | 0.618 |
| GBLUP+BLASSO | 0.612 | 0.567 | 0.575 | 0.601 | 0.635 | 0.615 |
| RKHS | 0.606 | 0.567 | 0.572 | 0.598 | 0.624 | 0.610 |
| RKHS+BayesA | 0.614 | 0.575 | 0.583 | 0.610 | 0.65 | 0.625 |
| RKHS+BayesB | 0.615 | 0.576 | 0.584 | 0.611 | 0.652 | 0.627 |
| RKHS+BayesC | 0.613 | 0.573 | 0.581 | 0.610 | 0.637 | 0.622 |
| RKHS+BLASSO | 0.610 | 0.571 | 0.577 | 0.605 | 0.636 | 0.617 |

**Table 2** Average accuracy of different prediction models for different training population sizes in a soybean nested association mapping population. Models include additive methods (BayesA, BayesB, BayesC, BLASSO), kernel methods (GBLUP, RKHS) and each combination of both.

|  | 250 | 500 | 1000 | 2000 | 3000 | 4000 |
| --- | --- | --- | --- | --- | --- | --- |
| BayesA | 0.386 | 0.491 | 0.603 | 0.685 | 0.723 | 0.749 |
| BayesB | 0.389 | 0.496 | 0.604 | 0.685 | 0.724 | 0.748 |
| BayesC | 0.385 | 0.492 | 0.601 | 0.680 | 0.719 | 0.744 |
| BLASSO | 0.374 | 0.481 | 0.594 | 0.680 | 0.721 | 0.747 |
| GBLUP | 0.374 | 0.481 | 0.593 | 0.676 | 0.716 | 0.743 |
| GBLUP+BayesA | 0.387 | 0.494 | 0.603 | 0.685 | 0.723 | 0.749 |
| GBLUP+BayesB | 0.390 | 0.496 | 0.606 | 0.686 | 0.724 | 0.749 |
| GBLUP+BayesC | 0.386 | 0.492 | 0.600 | 0.680 | 0.719 | 0.745 |
| GBLUP+BLASSO | 0.379 | 0.486 | 0.595 | 0.679 | 0.720 | 0.747 |
| RKHS | 0.374 | 0.485 | 0.593 | 0.672 | 0.711 | 0.741 |
| RKHS+BayesA | 0.389 | 0.497 | 0.605 | 0.686 | 0.725 | 0.753 |
| RKHS+BayesB | 0.391 | 0.499 | 0.607 | 0.687 | 0.726 | 0.754 |
| RKHS+BayesC | 0.388 | 0.495 | 0.602 | 0.681 | 0.720 | 0.749 |
| RKHS+BLASSO | 0.380 | 0.490 | 0.597 | 0.678 | 0.720 | 0.751 |

**Table 3** Average accuracy of different prediction models for 3 genotyping densities in a soybean nested association mapping population. Models include additive methods (BayesA, BayesB, BayesC, BLASSO), kernel methods (GBLUP, RKHS) and each combination of both.

|  | 1020 | 2039 | 4077 |
| --- | --- | --- | --- |
| BayesA | 0.601 | 0.606 | 0.611 |
| BayesB | 0.602 | 0.608 | 0.613 |
| BayesC | 0.600 | 0.605 | 0.607 |
| BLASSO | 0.595 | 0.600 | 0.603 |
| GBLUP | 0.593 | 0.599 | 0.600 |
| GBLUP+BayesA | 0.602 | 0.608 | 0.611 |
| GBLUP+BayesB | 0.603 | 0.609 | 0.613 |
| GBLUP+BayesC | 0.600 | 0.605 | 0.607 |
| GBLUP+BLASSO | 0.597 | 0.602 | 0.604 |
| RKHS | 0.592 | 0.598 | 0.598 |
| RKHS+BayesA | 0.605 | 0.611 | 0.613 |
| RKHS+BayesB | 0.606 | 0.612 | 0.615 |
| RKHS+BayesC | 0.601 | 0.608 | 0.609 |
| RKHS+BLASSO | 0.599 | 0.604 | 0.605 |

**Table 3** Average accuracy of 3 genotyping densities across population size in a soybean nested association mapping population.

|  | 1020 | 2039 | 4077 |
| --- | --- | --- | --- |
| 250 | 0.388 | 0.388 | 0.375 |
| 500 | 0.496 | 0.497 | 0.480 |
| 1000 | 0.603 | 0.602 | 0.596 |
| 2000 | 0.682 | 0.680 | 0.683 |
| 3000 | 0.722 | 0.723 | 0.717 |
| 4000 | 0.739 | 0.757 | 0.747 |

**Table 4** Average predictive ability (standard deviation) of 3 prediction models including BayesB (BB), GBLUP (G) and the combination of both, for grain yield in two years, in a 10-fold cross-validation scheme repeated 20 times, for each SoyNAM family.

| Family | 2013 | | | 2014 | | | |
| --- | --- | --- | --- | --- | --- | --- | --- |
|  | BB | G | BB+G |  | BB | G | BB+G |
| NAM2 | 0.318 (0.212) | 0.337 (0.207) | 0.322 (0.200) |  | 0.545 (0.199) | 0.549 (0.201) | 0.542 (0.210) |
| NAM3 | 0.385 (0.209) | 0.359 (0.218) | 0.368 (0.215) |  | 0.559 (0.178) | 0.534 (0.190) | 0.551 (0.177) |
| NAM4 | 0.246 (0.269) | 0.245 (0.262) | 0.259 (0.263) |  | 0.134 (0.223) | 0.135 (0.226) | 0.141 (0.223) |
| NAM5 | 0.358 (0.186) | 0.372 (0.202) | 0.360 (0.191) |  | 0.106 (0.369) | 0.107 (0.363) | 0.092 (0.374) |
| NAM6 | 0.103 (0.209) | 0.135 (0.230) | 0.110 (0.213) |  | 0.359 (0.150) | 0.380 (0.153) | 0.359 (0.154) |
| NAM8 | 0.258 (0.246) | 0.283 (0.234) | 0.249 (0.247) |  | 0.433 (0.225) | 0.423 (0.222) | 0.427 (0.221) |
| NAM9 | 0.152 (0.247) | 0.154 (0.258) | 0.157 (0.242) |  | 0.456 (0.252) | 0.473 (0.243) | 0.459 (0.241) |
| NAM10 | 0.330 (0.181) | 0.329 (0.155) | 0.338 (0.160) |  | 0.465 (0.182) | 0.464 (0.181) | 0.461 (0.189) |
| NAM11 | 0.506 (0.180) | 0.520 (0.178) | 0.503 (0.187) |  | 0.336 (0.163) | 0.345 (0.158) | 0.337 (0.179) |
| NAM12 | 0.556 (0.160) | 0.549 (0.158) | 0.558 (0.163) |  | 0.136 (0.251) | 0.112 (0.253) | 0.134 (0.242) |
| NAM13 | 0.315 (0.248) | 0.309 (0.239) | 0.317 (0.250) |  | 0.451 (0.154) | 0.432 (0.162) | 0.443 (0.163) |
| NAM14 | 0.292 (0.218) | 0.300 (0.207) | 0.301 (0.212) |  | 0.224 (0.197) | 0.203 (0.207) | 0.243 (0.206) |
| NAM15 | 0.246 (0.231) | 0.257 (0.235) | 0.238 (0.234) |  | 0.280 (0.213) | 0.268 (0.217) | 0.263 (0.214) |
| NAM17 | 0.296 (0.288) | 0.287 (0.287) | 0.300 (0.290) |  | 0.453 (0.157) | 0.419 (0.153) | 0.442 (0.150) |
| NAM18 | 0.199 (0.324) | 0.211 (0.303) | 0.195 (0.321) |  | 0.075 (0.273) | 0.089 (0.259) | 0.071 (0.278) |
| NAM22 | 0.299 (0.228) | 0.279 (0.209) | 0.304 (0.221) |  | 0.250 (0.271) | 0.242 (0.290) | 0.265 (0.274) |
| NAM23 | 0.350 (0.207) | 0.319 (0.219) | 0.333 (0.212) |  | 0.374 (0.248) | 0.370 (0.227) | 0.369 (0.251) |
| NAM24 | 0.297 (0.225) | 0.263 (0.217) | 0.274 (0.222) |  | 0.455 (0.141) | 0.430 (0.155) | 0.455 (0.142) |
| NAM25 | 0.619 (0.121) | 0.615 (0.113) | 0.620 (0.115) |  | 0.001 (0.332) | 0.029 (0.315) | 0.001 (0.327) |
| NAM26 | 0.288 (0.178) | 0.249 (0.189) | 0.271 (0.179) |  | 0.128 (0.291) | 0.138 (0.270) | 0.127 (0.301) |
| NAM27 | 0.372 (0.229) | 0.372 (0.216) | 0.382 (0.228) |  | 0.195 (0.279) | 0.194 (0.284) | 0.199 (0.288) |
| NAM28 | 0.093 (0.317) | 0.077 (0.297) | 0.106 (0.320) |  | 0.182 (0.333) | 0.179 (0.311) | 0.184 (0.329) |
| NAM29 | 0.204 (0.166) | 0.240 (0.154) | 0.224 (0.165) |  | -0.127 (0.251) | -0.148 (0.250) | -0.116 (0.289) |
| NAM30 | 0.106 (0.233) | 0.084 (0.248) | 0.106 (0.239) |  | 0.356 (0.198) | 0.344 (0.197) | 0.367 (0.203) |
| NAM31 | 0.189 (0.335) | 0.226 (0.334) | 0.176 (0.316) |  | 0.298 (0.233) | 0.306 (0.234) | 0.301 (0.241) |
| NAM32 | 0.018 (0.267) | 0.032 (0.269) | 0.026 (0.278) |  | -0.039 (0.284) | -0.042 (0.303) | -0.036 (0.285) |
| NAM33 | 0.183 (0.207) | 0.182 (0.207) | 0.176 (0.218) |  | -0.070 (0.246) | -0.066 (0.226) | -0.079 (0.238) |
| NAM34 | 0.264 (0.295) | 0.268 (0.291) | 0.260 (0.297) |  | 0.383 (0.166) | 0.361 (0.175) | 0.385 (0.164) |
| NAM36 | 0.016 (0.274) | -0.014 (0.264) | 0.016 (0.263) |  | -0.035 (0.374) | -0.022 (0.355) | -0.042 (0.356) |
| NAM37 | 0.177 (0.245) | 0.199 (0.228) | 0.184 (0.230) |  | 0.155 (0.277) | 0.161 (0.284) | 0.168 (0.280) |
| NAM38 | 0.255 (0.305) | 0.201 (0.321) | 0.256 (0.318) |  | 0.382 (0.229) | 0.406 (0.212) | 0.377 (0.240) |
| NAM39 | 0.163 (0.207) | 0.113 (0.217) | 0.177 (0.183) |  | 0.213 (0.261) | 0.248 (0.278) | 0.240 (0.253) |
| NAM40 | 0.100 (0.295) | 0.110 (0.288) | 0.087 (0.275) |  | 0.087 (0.282) | 0.100 (0.276) | 0.088 (0.274) |
| NAM41 | 0.352 (0.292) | 0.350 (0.298) | 0.360 (0.296) |  | 0.314 (0.234) | 0.315 (0.220) | 0.319 (0.231) |
| NAM42 | 0.357 (0.283) | 0.369 (0.268) | 0.356 (0.272) |  | 0.196 (0.315) | 0.218 (0.291) | 0.205 (0.313) |
| NAM46 | -0.012 (0.244) | -0.002 (0.252) | -0.016 (0.254) |  | 0.123 (0.225) | 0.144 (0.221) | 0.129 (0.233) |
| NAM48 | 0.157 (0.246) | 0.190 (0.252) | 0.152 (0.239) |  | 0.237 (0.223) | 0.196 (0.233) | 0.192 (0.247) |
| NAM50 | 0.170 (0.296) | 0.145 (0.321) | 0.173 (0.311) |  | 0.197 (0.246) | 0.191 (0.266) | 0.199 (0.262) |
| NAM54 | 0.124 (0.188) | 0.130 (0.173) | 0.129 (0.182) |  | 0.152 (0.237) | 0.171 (0.231) | 0.148 (0.242) |
| NAM64 | 0.024 (0.138) | 0.002 (0.146) | 0.035 (0.143) |  | 0.194 (0.268) | 0.185 (0.278) | 0.209 (0.259) |
